# Deciphering the Role of the Coagulation Cascade and Autophagy in Cancer-Related Thrombosis and Metastasis

**DOI:** 10.3389/fonc.2020.605314

**Published:** 2020-12-07

**Authors:** Charlotte Nicole Hill, Maria Paz Hernández-Cáceres, Catalina Asencio, Begoña Torres, Benjamin Solis, Gareth I. Owen

**Affiliations:** ^1^ Faculty of Biological Sciences, Pontificia Universidad Católica de Chile, Santiago, Chile; ^2^ Advanced Center for Chronic Diseases (ACCDiS), Santiago, Chile; ^3^ Millennium Institute on Immunology and Immunotherapy, Santiago, Chile; ^4^ Faculty of Medicine, Pontificia Universidad Católica de Chile, Santiago, Chile

**Keywords:** cancer, metastasis, coagulation, autophagy, cancer-associated thrombosis, megakaryopoeiesis and thrombopoiesis, protease (proteinase)-activated receptor

## Abstract

Thrombotic complications are the second leading cause of death among oncology patients worldwide. Enhanced thrombogenesis has multiple origins and may result from a deregulation of megakaryocyte platelet production in the bone marrow, the synthesis of coagulation factors in the liver, and coagulation factor signaling upon cancer and the tumor microenvironment (TME). While a hypercoagulable state has been attributed to factors such as thrombocytosis, enhanced platelet aggregation and Tissue Factor (TF) expression on cancer cells, further reports have suggested that coagulation factors can enhance metastasis through increased endothelial-cancer cell adhesion and enhanced endothelial cell activation. Autophagy is highly associated with cancer survival as a double-edged sword, as can both inhibit and promote cancer progression. In this review, we shall dissect the crosstalk between the coagulation cascade and autophagic pathway and its possible role in metastasis and cancer-associated thrombosis formation. The signaling of the coagulation cascade through the autophagic pathway within the hematopoietic stem cells, the endothelial cell and the cancer cell are discussed. Relevant to the coagulation cascade, we also examine the role of autophagy-related pathways in cancer treatment. In this review, we aim to bring to light possible new areas of cancer investigation and elucidate strategies for future therapeutic intervention.

## Introduction

**Figure 1:**
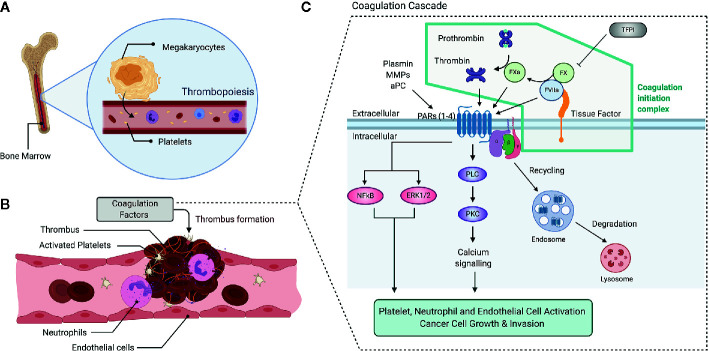
Overview of the coagulation and clotting factor signaling. Increased levels of platelet blood counts and coagulation cascade activation are determinants of a hypercoagulable state. Platelet production (thrombopoiesis) at the bone marrow by megakaryocytes determines platelet counts **(A)**. Upon vascular damage, platelets become activated, adhere to the vascular endothelium and promote neutrophil activation and NET release **(B)**. Platelets, Neutrophils and Endothelial cells become activated and promote thrombus formation, a process mediated by the coagulation cascade activation **(C)**. TF, a transmembrane receptor, initiate the extrinsic blood coagulation pathway once bound in a quaternary complex with FVIIa, that is inhibited by TFPI. The cleavage of FX by FVIIa gives rise to FXa, which in turn cleaves prothrombin into thrombin. Thrombin, FXa and other proteases such as Plasmin, MMPs, and aPC can activate PARs, which signal through PLC-PKC-Ca^2+^ Pathway and activate other signaling pathways such as NFkB and ERK1/2, leading to platelet, neutrophil and EC activation. This pathway also promotes cancer cell growth and invasion. PAR activation leads to receptor internalization, which classically occurs through the endolyosomoal pathway. aPC, activated Protein C; ERK1/2, Extracellular signal-regulated protein kinases 1 and 2; EC, Endothelial Cell; FVIIa, activated factor VII; FX, factor X; FXa, activated FX; MMPs, matrix metalloproteases; NET, Neutrophil Extracellular Trap; NFkB, Nuclear-Factor kappa B; PAR, protease activated receptor; TFPI, Tissue Factor Pathway Inhibitor.

While the precise role of the extrinsic coagulation cascade in the pathophysiology of cancer progression is still largely unknown, a hypercoagulable state has been intimately linked to cancer progression for more than a century (1). Cancer-associated thrombosis (CAT), or Trousseau’s syndrome, is attributed as the second leading cause of cancer patient death after organ failure upon metastatic disease (2, 3). High D-dimer levels, a product of coagulation cascade activation, are associated with advance cancer stage and accordingly there is a high prevalence of venous thromboembolism (VTE) in stage IV cancer patients (4–6). Given the risk of VTE being 4- to 7-fold higher in the cancer patient, modulation of the coagulation system is currently an essential aspect of cancer treatment (7), with the potential to improve in the coming years. A chronic hypercoagulable state in oncologic patients may have a multifaceted role in conferring both a survival advance and dissemination potential to a bourgeoning tumor. This hypercoagulable state can be explained by several factors, including increased megakaryocyte (MK) and platelet production (**Figure 1A**), increased platelet activation and deregulation of other cells intertwined with coagulation systems such as endothelial cells (ECs) and neutrophils (**Figure 1B**).

Different cell types express Tissue Factor (TF), including cancer cells, platelets, and activated endothelial and immune cells. The antibody blocking of TF has been shown to delay the initiation of tumor formation, growth and vascularization in immunodeficient mice (8). Collectively, data suggest that oncogenic and differentiation pathways regulate TF and that it functions in tumor initiation, tumor growth, angiogenesis, and metastasis. At the molecular level, TF, a transmembrane receptor belonging to cytokine receptor family, initiates the extrinsic blood coagulation pathway once bound in a quaternary complex with activated factor VII (FVIIa) and coagulation factor X (FX) (**Figure 1C**) [reviewed in (9)]. The cleavage of FX by FVIIa gives rise to the active protease (FXa), which in turn cleaves prothrombin to thrombin. This coagulation initiation complex can also directly activate cofactor VIII and coagulation factor IX, leading to thrombin generation (9).

The principal mediators of FXa and Thrombin protease signal transduction are the protease-activated receptors (PARs). These receptors belong to the G protein-coupled receptor (GPCR) family and become activated upon proteolytic cleavage of their N-terminal domain by extracellular proteases. PAR signaling has been implicated in several inflammatory diseases, including cancer (10, 11). Mammalian genomes contain four PARs that are ubiquitous within the body. PAR1, PAR3, and PAR4 are thrombin receptors, unlike PAR2. Classically, soluble proteases that are active during vascular injury, coagulation, and inflammation are responsible for PAR activation (12). Among these proteases are plasmin, matrix metalloproteinases (MMPs), activated Protein C (aPC), FVIIa, and FXa. However, the mechanisms remain elusive (13). The proteolytic cleavage of PARs activates numerous downstream signaling pathways, including intracellular Ca^2+^ mobilization, ERK1/2, NFκB signaling pathways and the induction of cytokines such as Interleukin-8 (IL-8) and IL-6 (14), allowing the PARs to mediate several processes in the coagulation-inflammation interplay, including those implicated in cancer progression (15, 16). Thus, PARs regulate platelet activation, assist in maintaining vascular barrier function through adhesion molecules (17, 18), and are associated with immune cell activation and migration (19–21). Upon activation, PARs are rapidly uncoupled from heterotrimeric G proteins through internalization to endosomes and then sorted to lysosomes and degraded. However, recent studies indicate that activated internalized PARs signal from endosomes through the recruitment of β-arrestins and potentially other pathways [reviewed in (10, 22)]. Interestingly, crosstalk between PAR signaling and autophagy has been described in different cell types (23–25), the physiological and pathophysiological role of this crosstalk still is an open field for investigation and discussion.

Autophagy, which comes from the Greek and means “self-eating”, is a highly regulated catabolic pathway by which cytoplasmic cargo is delivered to lysosomes for degradation and recycling, in order to preserve cellular homeostasis (26–29). In cancer, autophagy is broadly recognized as a “double-edged sword”, participating in both cancer suppression and promotion depending on tumor type, stage and microenvironment (30–32). Three principal types of autophagy have been identified: macroautophagy, microautophagy and chaperone-mediated autophagy (33, 34). Macroautophagy hereafter referred to as autophagy, is the most extensively characterized in cancer context and will be the focus of discussion within this review (35–37).

**Figure 2:**
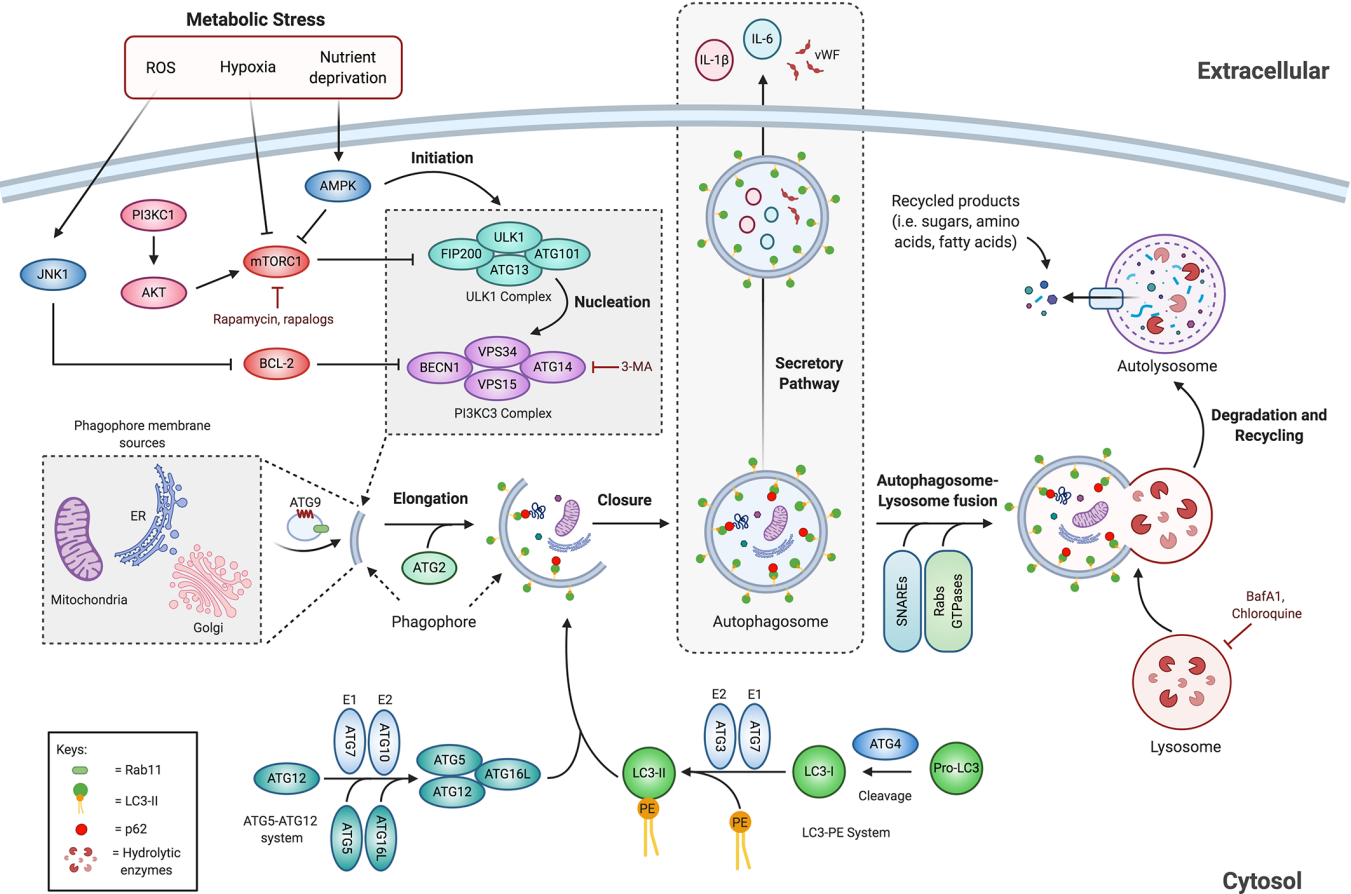
Overview of the autophagic pathway. Autophagy is induced by different stress stimuli including nutritional status, hypoxia and ROS. The autophagic process is initiated by the activity of ULK1 complex, which is regulated negatively by mTORC1 and positively by AMPK. ULK1 complex initiates phagophore nucleation by phosphorylating components of the PI3KC3 complex that leads to the recruitment of several ATGs to assist autophagosome formation. The elongation step involves two ubiquitin-like conjugation complex, the ATG5-ATG12 complex and the LC3-PE complex, which are required for proper phagophore membrane expansion and subsequent closure of the autophagosome. Completed autophagosome fuses with the lysosome to form the autolysosome, where hydrolytic enzymes degrade the enclosed material. The degrading metabolites are transported back to the cytosol for macromolecule synthesis and energy production. In addition, the autophagic machinery is associated with unconventional secretory processes. See the text for additional information. AMPK, AMP-activated protein kinase; ATG, autophagy-related proteins; BafA1, bafilomycin A1; BECN1, Beclin-1; BCL-2, B Cell Lymphoma 2; CQ, chloroquine; ER, endoplasmic reticulum; FIP200, focal adhesion kinase family interacting protein of 200 kDa; JNK1, c-Jun N-terminal kinase 1; LC3, microtubule-associated protein 1A/1B-light chain 3; mTORC1, mammalian target of rapamycin complex 1; PE, phosphatidylethanolamine; PI3K, phosphatidylinositol 3-kinase; SNAREs, soluble N-ethylmaleimide-sensitive factor attachment protein receptors; ULK1, unc-51-like kinase; VPS, vacuolar protein sorting; vWF, von Willebrand factor; 3-MA, 3-methyladenine.

Autophagy involves the sequestration of cytoplasmic material, such as damaged organelles (i.e. mitochondria, endoplasmic reticulum) and protein aggregates, in a double membrane organelle called the autophagosome, which subsequently fuses to lysosomes, forming a new vesicle known as the autolysosome. Lysosomal enzymes within the autolysosome initiate the hydrolytic breakdown of their cargo (30). The resulting degradation products (i.e., sugars, amino acids, fatty acids, nucleosides/nucleotides) are transferred back to the cytoplasm for macromolecule synthesis and energy production (**Figure 2**) (38). Therefore, autophagy sustains cellular homeostasis by regulating the quality of cytoplasmic organelles. Moreover, this is also an adaptive mechanism that promotes cell survival in response to stress conditions such as nutrient deprivation, hypoxia, reactive oxygen species (ROS), DNA damage, intracellular pathogens or an increase in misfolded proteins (39, 40). Cancer cells can exploit this mechanism when exposed to metabolic, oxidative and inflammatory stress (41–43). Different “autophagy-related proteins” (ATGs) participate in the autophagic process (44), which can be divided into different stages: initiation, nucleation, elongation, autophagosome maturation and fusion with the lysosome, and finally cargo degradation followed by the release of breakdown products into the cytosol.

As schematically represented in **Figure 2**, the “initiation stage” involves the activation of the ULK1 complex, which is composed of the Unc-51-Like Kinase 1 (ULK1), the Focal adhesion-kinase family Interacting Protein of 200 kDa (FIP200), ATG101, and ATG13. The ULK1 complex integrates nutrient and energy stress signals through the activity of the Serine/Threonine kinase Mammalian Target of Rapamycin Complex 1 (mTORC1), which is known as the master negative regulator of autophagy (29). In parallel, an isolation membrane called phagophore is formed by membrane contributions from various organelles, including the endoplasmic reticulum (ER), Golgi apparatus, and mitochondria (45). Once activated, the ULK1 complex translocates to a membranous site where the phagophore is formed, where it serves as a scaffold for the recruitment of ATG proteins to the isolation membrane and thereby initiating autophagosome biogenesis (46, 47).

In the following step, referred to as the “nucleation stage” (**Figure 2**), the ULK1 complex phosphorylates the class III Phosphatidylinositol 3-Kinase (PI3K) complex, composed of Vacuolar Sorting Protein 34 (VPS34), Beclin-1 (BECN1), VPS15, and Autophagy Related 14-Like protein (ATG14L). The class III PI3K complex promotes local production of phosphatidylinositol 3-phosphate (PI3P) at the phagophore and initiates the recruitment of effector proteins to assist autophagosome formation (48, 49). ATG9, the only transmembrane protein that is part of the ATG machinery, cycles between the phagophore and the Golgi/endosomes, contributing to the recruitment of membranes for the formation and subsequent expansion of the phagophore (47, 50, 51). The sorting of ATG9 and its following transport to phagophore membranes occurs at RAB11-positive recycling endosomes (52, 53). Besides, ATG2 participates in the transfer of lipids from the ER to the phagophore leading to its expansion (54, 55).

The subsequent step is the “elongation stage” and involves the extension of the phagophore membrane (**Figure 2**). This process requires two ubiquitin-like systems: the ATG5–ATG12 system and the Microtubule-Associated Protein 1A/1B-Light Chain 3 (MAP1LC3A/B, also known as LC3)-phosphatidylethanolamine (PE) system (56). First, ATG12 is conjugated to ATG5 by the sequential activity of ATG7 and ATG10. The resulting ATG5-ATG12 complex interacts with ATG16L, leading to the formation of the multimeric complex ATG5-ATG12-ATG16L which is fundamental for LC3 lipidation (57, 58). Second, pro-LC3 is cleaved by ATG4 to form the cytosolic soluble LC3-I. Subsequently, ATG7 and ATG3 activity enable LC3-I conjugation to the lipid PE to form LC3-II, which is then recruited to the inner and outer surface of the autophagosome in an ATG5-ATG12 dependent manner (59). This process elongates and seals the phagophore leading to the subsequent formation of the autophagosome. Importantly, under basal conditions, LC3-I is uniformly distributed across the cytoplasm; however, upon autophagy induction, the lipidated form of LC3 (LC3-II) accumulates on nascent autophagosomes, and thus, represents a marker to monitor the formation of autophagosomes and autophagy-related structures (60–62). Autophagic cargo selection occurs in parallel to the processes of sensing, initiation and elongation. Cargo adaptors such as the receptor protein Sequestosome 1/p62 (SQSTM1/p62) can interact with both ubiquitin chains and LC3, and thereby promote ubiquitinated cargo recruitment to autophagosomes for selective degradation (63). As p62 becomes incorporated within the autophagosomes and are degraded in autolysosomes, thus serving as an index of autophagic degradation (60). Upon closure, the autophagosome dissociates from the assembly site and undergoes “maturation” *via* fusion with endosomes and subsequently with lysosomes to form a degradative autolysosome (64, 65). Maturation and autophagosome-lysosome fusion requires several proteins including Rab GTPases, membrane-tethering complexes and soluble N-ethylmaleimide-sensitive factor attachment protein receptors (SNAREs) (66–68).

Finally, the lysosomal hydrolases degrade the autophagic cargo, and the resulting metabolites get recycled and returned to the cytosol through autolysosome efflux transporters, and thus cellular homeostasis is maintained (34, 69, 70).

Autophagy is highly regulated by different signaling pathways implicated in cancer (36, 71, 72). Nutrient starvation is the best-characterized autophagy inductor, where the serine/threonine protein kinase mTOR plays a critical role as an energy sensor (73). Within the human cell, mTOR can be found in at least two distinct multiprotein complexes, referred to as mTOR complex 1 (mTORC1) and mTOR complex 2 (mTORC2) (74). The mTORC1 complex is considered the primary negative regulator of autophagy (75, 76). Under nutrient-rich conditions, class I PI3K and AKT/PKB activate mTORC1 complex which by phosphorylating ULK1 and ATG13, prevents the induction of autophagy as shown in **Figure 2** (77–80). A sensor of available energy is the AMP-activated protein kinase (AMPK), which is directly activated by a low ATP:ADP ratio (81, 82). Under starving, AMPK directly phosphorylates and inactivates mTORC1 (83). Through AMPK regulation, the inhibition of mTORC1 and the activation of the ULK complex can initiate the autophagy process (**Figure 2**) (46, 77).

Numerous factors that regulate autophagy are also classified as either oncoproteins or products of tumor suppressor genes [reviewed in (36, 71, 84)]. Thus, autophagy-signaling pathways are caught up in cancer regulation and control (**Figure 2**). Oncoproteins, including the small GTPase RAS, RHEB, and Nuclear Factor-κB (NF-κB), can activate mTORC1 and in consequence inhibit autophagy (85). NF-κB activates autophagy by inducing the expression of proteins involved in autophagosome formation, including BECN1, ATG5, and LC3. Conversely, NF-κB can also inhibit the autophagic process by increasing the expression of autophagy repressors, such like B cell lymphoma 2 (Bcl-2) family members (86). The anti-apoptotic members of the B Cell Lymphoma 2 (Bcl-2)-family bind and sequester BECN1 to prevent autophagy induction (87). On the contrary, tumor suppressors such as the transcription factor Forkhead box O1 (FOXO1) and nuclear p53 are known to induce autophagy (88). Interestingly, ROS production, a hallmark of cancer, and the subsequent activation of the oncogene c-Jun N-terminal kinase1 (JNK1) (89) can lead to the phosphorylation of Bcl-2; this prevents the interaction of this latter protein with BECN1 and thereby induces autophagy (88).

Pharmacological agents are frequently used to either enhance or suppress autophagy (**Figure 2**) (90). A frequent used approach for autophagy induction is mTOR inhibition by rapamycin (91). Conversely, 3-methyladenine (3-MA) can suppress the nucleation stage by inhibiting the PI3K complex, thereby inhibiting autophagosome formation (92). Autophagy can be blocked at later stages resulting in the inhibition of autophagic flux. This refers to the entire process from autophagosome synthesis to lysosomal degradation (93). Bafilomycin A1 (BafA1) is a potent V-ATPase inhibitor that impairs lysosomal acidification and thus the degradation of autophagic cargo (94). By a similar approach, chloroquine (CQ) can inhibit autophagy by increasing the lysosomal pH and therefore reducing the activity of degradative enzymes (95). Accordingly, BafA1 and CQ are commonly used to decrease the autophagic flux.

Although canonically characterized as a degradation mechanism, recent evidence has demonstrated a role for the autophagic machinery in extracellular secretion, a process termed as “secretory autophagy” or more linguistically precise “ATG gene-dependent secretion” (96–98). Accordingly, canonical autophagy involves the fusion of the autophagosomes with lysosomes for cargo degradation, whereas the secretory pathway bypasses this degradative process to allow unconventional extracellular delivery of cytosolic proteins *via* LC3-positive vesicles (**Figure 2**) (99, 100). Even though the molecular pathways in secretory autophagy are not entirely deciphered, the molecular machinery of the degradative processes is required (99). ATG5 and BCN1, together with other factors participating in canonical autophagy, are also activated as part of the secretory pathway (98, 101). The secretory autophagy pathway plays a key role in the progression of several diseases, including cancer (102, 103). It is involved in the secretion of cytokines such as IL-6, IL-8, and IL-1β, damage response mediators such as High mobility group box 1 protein (HMGB1) or ATP, and granule content such as von Willebrand factor (vWF) (104–107). Particularly, autophagy-dependent secretion of IL-6 has been implicated in tumor cachexia (103) and the metastatic potential of Ras-transformed cancer cells (108). The secretory autophagy pathway is reported to mediate cytokine release from cancer-associated fibroblasts, contributing to the development of head and neck squamous cell carcinoma (102). Thus, within the TME, both the cancer cells and supporting stromal cells rely on autophagy-dependent secretion for malignant progression (98).

Interestingly, it has been observed that decreased autophagosome degradation is associated with increased release of extracellular vesicles (EVs) in human malignant cervical and breast cancer cell lines (109). Extracellular secretion may provide a supplementary pathway to maintain cellular homeostasis when the degradative autophagy pathway is blocked (109). These suggest possible crosstalk between degradative and secretory autophagy to maintain cellular homeostasis and tumor cell survival. Furthermore, following pathological stress, there is a reported release of cytosolic proteins, molecular chaperones, harmful nucleic acids and misfolded proteins into the extracellular space through EVs, and this may contribute to tumor progression and metastasis (110, 111).

There is a complex association between cancer and autophagy. While in late-stage disease autophagy promotes tumor progression by providing nutrients to a rapidly dividing yet under vascularized and undernourished tumor, at early stages autophagy may suppress the bourgeoning tumor by suppressing reactive oxygen species and thus limiting genomic instability and, furthermore, promoting an anti-inflammatory microenvironment (36, 112–115).

Noteworthy is that the participation of the coagulation cascade in cancer progression is not limited only to thrombogenesis and thrombocytosis. The hemostatic system is known to promote tumor growth and metastasis, with thrombin increasing proliferation, migration and angiogenesis in preclinical models (116, 117). Circulating Tumor Cells (CTCs) are a marker of poor prognosis and are associated with increased risk of VTE in cancer patients (6, 118). TF has been identified to promote and be present on cancer stem cells (119, 120). Moreover, cancer and adjacent cell expression of PAR1 was recently postulated as a predictive marker of metastasis (121). The plasmatic concentration of Thrombin–Anti-Thrombin III (TAT) complex, a surrogate marker for activated thrombin used to assess coagulation state, inversely correlates with cancer patient survival (6). Independently of a direct role in the clotting process, the TF-FVIIa-PAR2 signaling was also linked to breast and liver cancer progression (23, 122).

Mouse models of cancer have demonstrated that FXa can increase tumor growth (15, 19, 20). Moreover, FXa increased lung, lymph node, liver, kidney metastasis in a syngeneic melanoma model, while promoting vascular permeability and increased infiltration (15). How PAR activation impinges on the autophagic pathway and how this regulates both physiological and pathophysiological processes is still an area requiring further study. We discuss herein that the coagulation system is not merely a bystander in cancer metastasis, but instead an integral part of the multifaceted approach taken by the malignant cell to survive, propagate, and ultimately exhaust the body’s capacity to function. We speculate that the malignant cell may use common mechanisms to hijack immune cells, ECs, hematopoietic cells, and platelets to integrate with the coagulation system for its own end. In this review, we postulate that a common mechanism present in each of these components may be the hijacking of the autophagy pathway, given the reported correlation between CTCs, hypercoagulable state and reduced survival in cancer. We examine the evidence for shared mechanisms and pathway integration to better understand cancer pathophysiology and to uncover novel druggable targets for the oncology clinic.

## Autophagy Regulates Thrombus Formation Through Megakaryocyte Differentiation, Platelet Production, and Platelet Activation

### Autophagy Is Implicated in Megakaryopoiesis and Thrombopoiesis

**Figure 3:**
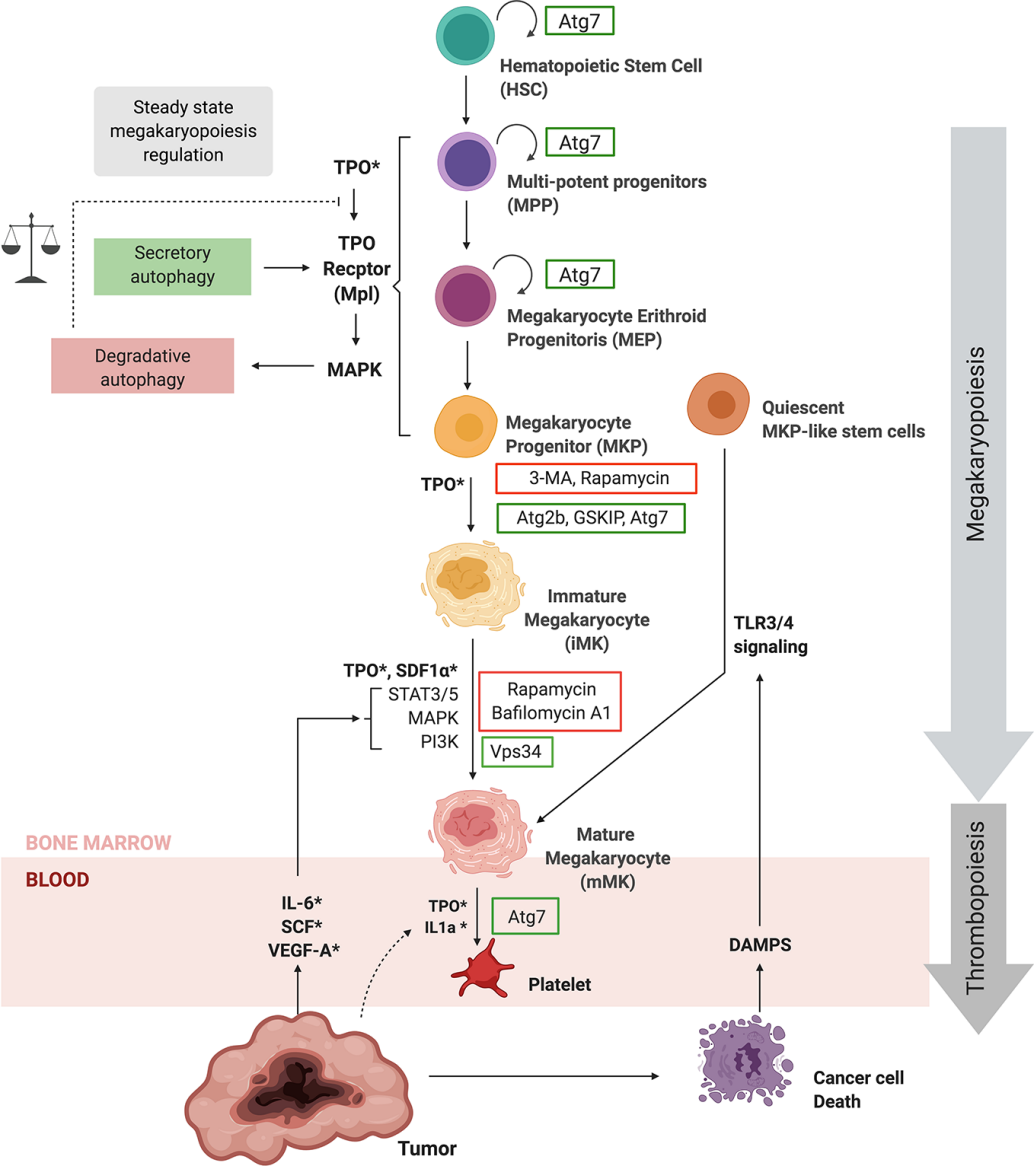
Autophagy regulates megakaryopoiesis and thrombopoiesis. Hematopoietic stem cells give origin to megakaryocytes (MKs) through megakaryopoiesis by a succession lineage commitment steps, followed by a maturation process. TPO is the main growth factor regulating steady-state megakaryopoiesis and thrombopoiesis, which bind to the Thrombopoietin receptor (Mpl). Autophagic regulation of the Thrombopoietin receptor plays a key role in steady-state megakaryopoiesis. ATG7, ATG2B, and GSKIB are necessary for megakaryopoiesis, while BafA1, CQ and rapamycin have an impact at different steps of the differentiation process. Furthermore VPS34 is also necessary for MK maturation. Inflammatory signaling through TLR/NFkB pathways promotes the differentiation of quiescent MKP–like stem cells into megakaryocytes during emergency megakaryopoiesis and thrombopoiesis upon injury. Importantly, cancer increases systemic levels of TPO, IL-1α, IL-6, SCF, VEGF-A, and DAMPs that could not only enhance steady state thrombopoiesis but also emergency megakaryopoiesis and thrombopoiesis. The regulation of autophagy during megakaryopoiesis and thrombopoiesis still requires further investigation. For more information please refer to text. ATG, Autophagy-Related Gene; BafA1, Bafilomycin A1; BECN1, Beclin-1; CQ, Chloroquine; DAMPs, Damage-Associated Molecular Patterns; IL-1α, Interleukin 1 alfa; IL-6, Interleukin 6; MAPK, Mitogen-Activated Protein Kinase; PI3K, Phosphatidylinositol 3-Kinase; STAT, Signal transducer and activator of transcription 3; Thrombopoietin, TPO; VPS, Vacuolar Protein Sorting; 3-MA, 3-methyladenine; SDF-1, stromal cell-derived factor 1.

The body produces 1 × 10^11^ platelets per day to maintain platelet count through the processes of megakaryopoiesis and thrombopoiesis (123). As schematically represented in **Figure 3**, within the bone marrow, the process of megakaryopoiesis gives origin to MKs from Hematopoietic Stem Cells (HSCs) through successive lineage commitment steps, followed by a maturation process. HSC differentiate through sequential steps into multipotent progenitors (MPPs), common myeloid progenitors (CMPs), bipotential MK-Erythroid progenitors (MEPs), and unipotent MK progenitors (MKPs), which then mature into MKs (124–127). The principal growth factor regulating steady-state megakaryopoiesis and thrombopoiesis is Thrombopoietin (TPO), which influences almost every step of the differentiation and maturation process. TPO is involved in HSC self-renewal, the proliferation of MKPs, MK maturation, and platelet production. TPO binds to the Thrombopoietin receptor (Mpl) activating JAK2 signaling, STAT3/5 and MAPK pathways and in this way positively regulates MK and platelet production (128).

Autophagy and megakaryocytic differentiation are overlapping processes. An up-regulation of BECN1 and LC3II precedes megakaryocytic differentiation. Moreover, autophagy is necessary for MK differentiation *in vitro* (129). Human CD34+ hematopoietic progenitor cell differentiation into MKs is slowed down by the autophagy inductor rapamycin, resulting in a lower percentage of and smaller MKs (130). It was further shown that ATG2B and the GSK3B-interacting protein (GSKIP) enhanced the differentiation of CD34+ progenitors and MKPs into MKs by increasing progenitor TPO sensitivity (131).

Moreover, it seems that autophagic flux regulation and its counterbalance of the apoptosis pathway is a crucial aspect during megakaryopoiesis (132). Interestingly, TPO stimulation results in TPO-Receptor internalization and targeting to the autolysosome for degradation (133, 134). Besides the canonical ER-Golgi route, the receptor traffics to the membrane through secretory autophagy (133, 134). Based on these studies, steady-state megakaryopoiesis and thrombopoiesis may be maintained by MAPK activation, which in turns targets the TPO receptor to autolysosomes and thus decreases TPO sensitivity in MPPs. However, an increase in p62 and thus disruption of the autophagic flux is observed (129), indicating that TPO could increase autolysosome formation but not necessarily degradation, thus favoring secretory autophagy. TPO activation of MAPK signaling may be promoting autophagy and therefore reducing TPO sensitivity; however, sensitivity could increase through secretory autophagy mediated by BECN1, ATG2B, and GSKIP (**Figure 3**). Therefore, the balance between degradative and secretory autophagy may play a key role in TPO sensitivity and steady-state megakaryopoiesis.

Further demonstrating the dependence of the autophagic pathway in steady-state megakaryopoiesis, VPS34, a protein required at the “nucleation stage”, is implicated in MK migration, Demarcation Membrane System (DMS) development, pro-platelet formation and platelet release (135, 134). Although rapamycin strongly inhibits polyploidization and pro-platelet formation (130, 137), hematopoietic lineage-specific *Atg7* deletion demonstrated that the autophagy machinery is necessary for thrombopoiesis (138). *Atg7* deletion not only impaired autophagy but also megakaryopoiesis, MK differentiation and thrombopoiesis. In particular, *Atg7* knockout (KO) mice presented fewer platelets and failed to maintain hemostasis (138). In contrast, a separate study using *Atg7* ^f/f^; PF4-Cre mice failed to demonstrate a role for *Atg7* in thrombopoiesis (139). Differing results may be explained by HSC relying exclusively on ATG7-dependent autophagy; however, differentiated cells may trigger compensatory non-canonical signaling pathways (140). Given the role of *Atg7* in the initiation of canonical autophagy, this process appears to be crucial for both MK and platelet production and function.

Under stress conditions such as injury and infections, platelets are rapidly consumed, representing a high risk for health. Inflammatory signaling leads to a 10-fold increase in platelet production and platelet size (141). As shown in **Figure 3**, the regulatory mechanism of thrombopoiesis is mainly attributed to IL-6 promoting TPO secretion from the liver (142). However, inflammatory signaling could have a more direct effect as STAT3 activation is required for MKP expansion, MK maturation, and platelet production in a TPO independent fashion (143). Further mechanisms may involve the potentiation of megakaryopoiesis through TLR-mediated activation of quiescent stem-like MK-committed progenitors and IL1a driven MK rapid cytoplasmic fragmentation (144, 145). While autophagy regulation by inflammatory stimuli is well studied (42, 97), to our knowledge, there are no reports to date implicating autophagy in inflammation-mediated thrombopoiesis.

Taken together, published data strongly suggest the relevance of the autophagic process during steady-state megakaryopoiesis. Interestingly, autophagic activity may vary between stages of commitment and differentiation and may be involved in TPO sensitivity and pro-platelet production in steady-state thrombopoiesis. The relationship between autophagy and inflammatory signaling, as well as the existence of inflammatory induced thrombopoiesis, raises the question if these processes are connected. Further studies are required to clarify the role of autophagy during megakaryopoiesis and thrombopoiesis.

### Crosstalk Between Coagulation Cascade and Autophagy During Platelet Activation

Despite being a developing field of research, the machinery and cellular structures of autophagy have been identified to be present and operative in platelets, indicating that platelets undergo basal autophagy much like nucleated cells. Autophagosome-like structures in platelets were first described during the 70s (146) and recently confirmed by super-resolution microscopy (147). Furthermore, the presence of autophagy-related structures and proteins are also evidenced in resting platelets (139, 147–150). Starvation or rapamycin treatment increases the autophagy in platelets in an ATG5-dependent fashion, which is reversed by 3-MA (151, 152). Although still under study, the physiological relevance of autophagy in platelets appears to be related to their activation. Firstly, autophagy can be induced during platelet activation by hemostatic agonists (139, 151). Secondly, defective platelet activation has been reported in *Atg7*-, *Atg5*-, *Becn1*-, *Vps34*- knockout mice (135, 139, 153).

**Figure 4:**
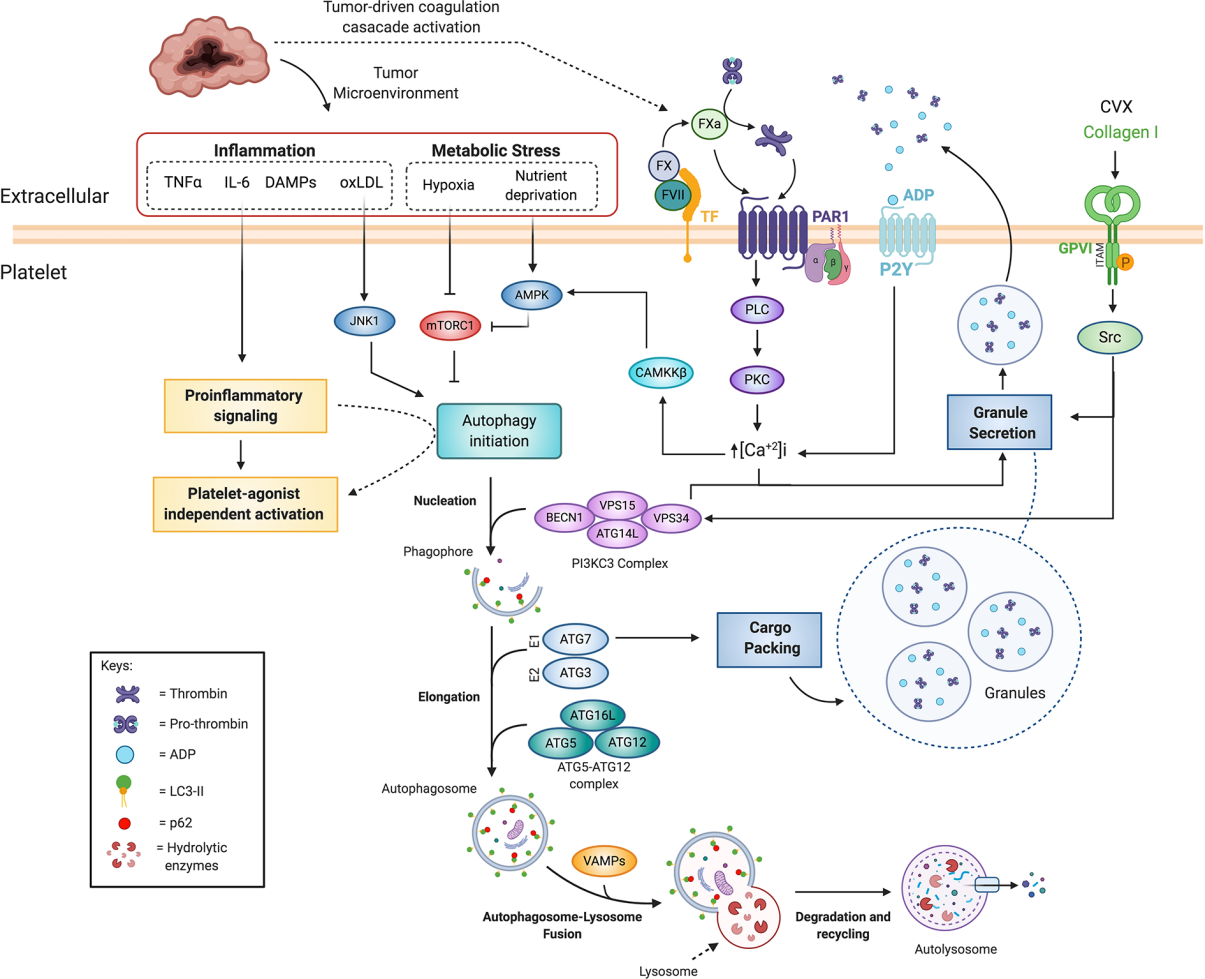
Coagulation cascade and autophagy crosstalk in platelet activation and cancer-associated thrombosis. Platelet agonists such as thrombin, ADP and collagen can increase autophagic flux, which is necessary for efficient platelet activation. PAR activation and P2Y engagement activate the PLC-PKC-Ca^2+^ signaling, which has been shown to increase platelet autophagic flux during platelet activation, possibly through CAMKKB. Additionally, autophagy proteins such as BCN1, VPS34, ATG7, ATG5, and VAMPs have been shown to be necessary for platelet activation, cargo packing and granule secretion. Tumors increase systemic inflammation and locally generate both hypoxia and nutrient deprivation, which cannot only promote platelet activation but also autophagy through JNK and AMPK activation, and mTORC1 inhibition. Pro-inflammatory signaling through increased levels of extracellular TNFα, IL-6, and IL-8, which are frequently elevated in cancer patient serum samples, is involved in both platelet activation and possibly modulating platelet autophagy. ADP, adenosine diphosphate; AMPK, AMP-activated protein kinase; ATG, autophagy-related proteins; BECN1, Beclin-1; CAMKKB, calcium/calmodulin-dependent protein kinase kinase 2; CVX, convulxin; DAMPs, Damage-Associated Molecular Patterns; FVII, Factor VII; FX, Factor X; FXa, activated FX; GPVI, Glycoprotein VI; IL-6, Interleukin 6; IL-8, Interleukin 8; JNK1, c-Jun N-terminal kinase 1; LC3, microtubule-associated protein 1A/1B-light chain 3; mTORC1, mammalian target of rapamycin complex 1; oxLDL, Oxidized low-density lipoprotein; PAR, protease activated receptor; PE, phosphatidylethanolamine; PI3K, phosphatidylinositol 3-kinase; PKC, Protein kinase C; PLC, Phospholipase C; TF,Tissue Factor; TNFα, Tumor Necrosis Factor α; VPS, vacuolar protein sorting; VAMP, Vesicle associated membrane proteins.

Platelet activation occurs upon collagen binding to platelet glycoproteins (such as GPVI), thrombin engagement of PARs, and ADP activation of P2X receptors [reviewed in (154)]. Activation of these pathways triggers positive feedback, with platelets releasing their granules containing more agonists such as prothrombin, ADP and among other growth factors (**Figure 4**). In parallel, integrins are upregulated on platelet membranes, allowing platelet aggregation and ultimately clot formation (155). Noteworthy, is that *Becn1* KO and platelet specific *Vps34* KO mice display defective collagen-induced platelet aggregation, adhesion and thrombus formation (136, 152, 153). This association still needs further investigation as *Becn1* is also dampened in ECs, among other cells relevant for hemostasis.

Interestingly, platelet specific *Vps34* KO showed impaired thrombus formation in two independent studies, indicating that platelet VPS34 is necessary for the clotting process (133 153). Accordingly, thrombin or C Reactive Protein (CRP) treatment significantly increases VPS34 dependent PI3P production in platelets while in *Vps34* ^−/−^ platelets and human platelets treated with 3-MA displayed impaired responses to thrombin, collagen and ADP (133). Furthermore, *Vps34* ^−/−^ platelets displayed impaired aggregation, dense granule secretion and decreased levels of Syk and PLCγ2 phosphorylation (153). In line with these findings, 3-MA impaired human platelet activation in response to Convulxin (a GPVI agonist) and thrombin, suggesting involvement of VPS34 in platelet activation downstream of PARs and GPVI (153). Although *Vps34* ^−/−^ platelets showed reduced mTOR signaling and increased LC3-II levels, the authors of this study did not demonstrate an association between autophagy and platelet activation (153). Alternatively, they proposed that VPS34 promotes PI3P–guided NADPH oxidase assembly and subsequent ROS generation, supporting PAR- and GPVI-mediated platelet activation (153). However, a separate study showed an increased rate of secretion in response to platelet agonists in VPS34-deficient platelets *ex vivo* and underflow conditions (133). Moreover, under arterial flow, VPS34-deficient platelets display an inefficiency in recruiting circulating WT platelets to the growing thrombus (133). These authors proposed that VPS34 production of PI3P contributes to the spatiotemporal regulation of granule secretion, possibly by recruitment of intracellular proteins that regulate granule fusion and secretion (133). Interestingly, ROS production in platelets suppresses the downstream activity of the PI3K/AKT/mTOR signaling pathway promoting autophagy and consequently exacerbating platelet aggregation (156). These observations raise the possibility that VPS34 mediation of ROS production may promote autophagy and subsequently platelet aggregation; however, this hypothesis still needs to be tested.

Interestingly, 65% of mice with a platelet specific deletion of *Atg7* (*Atg7* ^f/f^;*PF4-Cre*/^+^) were unable to arrest bleeding 10 min after tail transection despite no significant effects on platelet counts or volumes (139), further demonstrating that selective autophagy is an aspect for platelet activation. In the same line, it has been proposed that *Atg5* enables mitophagy in platelets, which is a requirement for correct platelet activation (157).

The concept that autophagy could be promoted downstream of platelet agonist signaling, and that in turns regulates platelet activation, is supported by the observation that autophagy inhibitors block platelet aggregation and adhesion. Pre-incubation of platelets with 3-MA, BafA1 or CQ is shown to inhibit Collagen I (COL1)- and thrombin-stimulated aggregation (151). Interestingly, thrombin reduces LC3II levels, indicating an increase of the autophagic flux upon platelet activation (139). Similarly, a PAR1 peptide and ADP both mediated a reduction in LC3II (139). LC3II reduction requires degradation through canonical autophagy which is mediated by key downstream elements of platelet activation signaling cascade, including phospholipase C, protein kinase C, Ca^2+^, and Src-family kinases, as shown in **Figure 4** (139) Although Src-family kinases seem to be upstream of LC3II increase in platelets, the specific mechanism has yet to be elucidated. On the other hand, extrapolating mechanisms reported in other cell types, Ca^2+^ may promote autophagy through CAMKKβ/AMPK/mTOR signaling (158). Hence, GPCR mediated platelet activation and subsequent increase in [Ca^2+^]_i_ could promote autophagy through this pathway (**Figure 4**). However, AMPK-independent Ca^2+^ regulation of autophagy has also been described (159). Thus, the possibility exists that Ca^2+^ promotes autophagy through a different mechanism.

The evidence of autophagy in resting platelets and, more importantly, of an increased autophagic flux regulation during platelet activation, clearly points to crosstalk between platelet agonists and autophagy and strongly suggests that autophagy is essential for coagulation. Furthermore, deficiency in key mediators of autophagosome formation such as VPS34 and ATG7 are associated with secretion and packaging of platelet granules, a fundamental aspect in thrombus formation. Taken together, platelet agonists signaling through PARs and GPVI could activate the autophagy signaling machinery, potentially mediated by Ca^2+^ and Src signaling, to promote autophagosome formation, which in turn promotes efficient granule packing and secretion (**Figure 4**).

### Autophagy Implication in Cancer-Associated Thrombocytosis Through the Regulation of Platelet Production and Activation

It is established that thrombocytosis at diagnosis correlates with enhanced tumor invasion, metastasis, and poor prognosis in several solid cancers. Mean platelet volume was recently proposed as a diagnostic biomarker for lung cancer (160), while platelet counts have been correlated with stage and survival in melanoma patients, were thrombocytosis at diagnosis was significantly associated with distant metastasis (161). The current line of thought is that tumors benefit from thrombocytosis through platelet interaction with the CTCs, the latter encapsulating the tumor cells protecting them from NK cells and promoting the maintenance of an epithelial to mesenchymal transition (EMT) state (119, 162–165).

Altered platelet production in cancer could be explained by inflammation-mediated thrombopoiesis. A hypothesis is that tumor production of IL-6 promotes megakaryopoiesis *via* hepatic TPO, leading to thrombocytosis (142, 166). However, other plasmatic thrombopoietic cytokines such as Stem Cell Factor (SCF), Interleukin 1 alpha (IL-1α), Tumor Necrosis Factor alpha (TNF-α), stromal cell-derived factor 1 (SDF1), and Vascular Endothelial Growth Factor A (VEGF-A) are increased in cancer patients, providing alternative pathways for thrombopoiesis that could be independent or complementary to the IL-6/TPO axis. The SDF1a-CXCR4 axis is independent of the TPO/Mpl axis in murine models (167). Moreover, VEGFR1 promotes MKP maturation, possibly through CCXR4 up-regulation, leading to increased platelet counts *in vivo* (168, 169). Likewise, VEGFR2 activation increases MK proliferation, survival and differentiation (170).

Moreover, it has been reported that quiescent MKPs can rapidly differentiate into mature MKs and replenish platelet counts in response to inflammatory stimuli (144). This may be triggered by malignant signaling, as Toll-Like Receptor (TLR) activation is implicated in both hematological and solid tumors (171). As previously discussed, autophagy inhibition in early megakaryopoiesis appears to impair MK maturation, reduce platelet formation, and affect platelet function. Although many of the thrombopoietic cytokines such as TPO, IL-6, TNF-α, and VEGF have been associated with autophagy in MK or other cell types (131, 172) it remains unknown if, through deregulation in cancer, autophagy is central to promoting platelet production and thus sustaining a vicious cycle between malignancy and thrombopoiesis.

Suggesting that platelet autophagy is somehow deregulated in cancer, Lewis et al. described an increase in autophagosome-like structures in platelets from cancer patients (146). In malignancy, an obvious path for platelet autophagy regulation and platelet activation is the deregulation of the coagulation-signaling cascade. As previously discussed, platelet agonists such as thrombin, ADP and collagen can increase autophagic flux which is necessary for efficient platelet activation. Pro-inflammatory cytokines such as TNFα, IL-6, and IL-8, which are frequently elevated in cancer patient serum samples (173), are also involved in both platelet activation and autophagy. TNFα was shown to promote the platelet activation independently of platelet agonists, increasing TF expression, thrombin generation and subsequent clot formation (174). Other pro-inflammatory cytokines such as IL-6 and IL-8 and NF-κ B activation can also promote platelet activation (175, 176). TLR, TNFα, NF-κB, and JAK/STAT signaling pathways have been widely reported to regulate autophagy (42, 172, 177); however, this has yet to be evaluated in platelets.

ROS regulates autophagy in various cells by modulating the PI3K/AKT/mTOR pathway, and recent studies show that this is also true in platelets (156). High blood levels of oxidized LDL (oxLDL) have been reported in cancer patients and associated with metastasis in breast, ovarian, gastric and prostate cancer (178–180). Noteworthy, these cancers are also associated with a higher risk for thrombosis (181). ox-LDL can increase platelet activation in a VPS34 dependent manner, suggesting the involvement of the autophagy process (156). In line with these findings, the Oxidized low-density lipoprotein receptor 1 (LOX-1), recognizes and binds activated platelets assisting in the formation of a thrombus (182).

Furthermore, a separate study revealed that autophagy was activated in platelets through an oxidative stress-induced JNK pathway, which was evidenced by increased co-localization of LC3II with the Lysosomal Associated Membrane Protein 1 (LAMP1), suggesting enhanced autolysosome formation, after hydrogen peroxide treatment (152). It was also observed that mitophagy reduced phosphorylated p53, thus preventing apoptosis, and conversely the absence of mitophagy resulted in increased thrombosis (152). In this way, tumor micro-environmental cues such as ROS could alter platelet selective autophagy of mitochondria and thereby regulate platelet activation and thrombus formation.

Taken together, tumor-derived soluble factors could promote thrombopoiesis and potentially deregulate autophagy in hematopoietic progenitors and MKs. TME factors may regulate autophagy and subsequent platelet activation, furthermore, a high coagulative state and platelet-derived factors may promote tumor growth, metastasis and chemotherapy resistance.

## Coagulation and Autophagy Cascade in Cancer Cells

TF and its protease products, such as Thrombin and FXa, are associated with cancer progression and cancer-related thrombosis (15, 20, 119, 183). Furthermore, this association may not be solely related to TF expression in the primary tumors as results have now shown that circulating microparticles carrying TF are 16- to 26-fold higher in pancreatic cancer patients with thrombosis when compared to healthy controls (183). There is emerging evidence that there is an involvement of the autophagy cascade in the mechanism controlling and promoting cancer progression and cancer-related thrombosis (23, 184). Proteins of the autophagy pathway are associated with cancer cell chemotherapy and radiation sensitivity through the alleviation of cellular stress and the dampening of apoptosis. Somewhat paradoxically, BECN1 has been postulated as a tumor suppressor due to its role in regulating p53 stability (185). As already mentioned, mTORC1 is the master negative regulator of the autophagic pathway and plays a critical role in cancer cell growth and progression (186, 187). Due to insufficient vascularization, tumors experience nutrient deprivation and hypoxia (188–190). In this scenario, mTORC1 may act as a restriction point between proliferation and differentiation (191). A hypoxic microenvironment also leads to an increase in both TF and FVII, correlating with tumor progression, local invasion, distant metastasis and therapeutic resistance (192).

**Figure 5:**
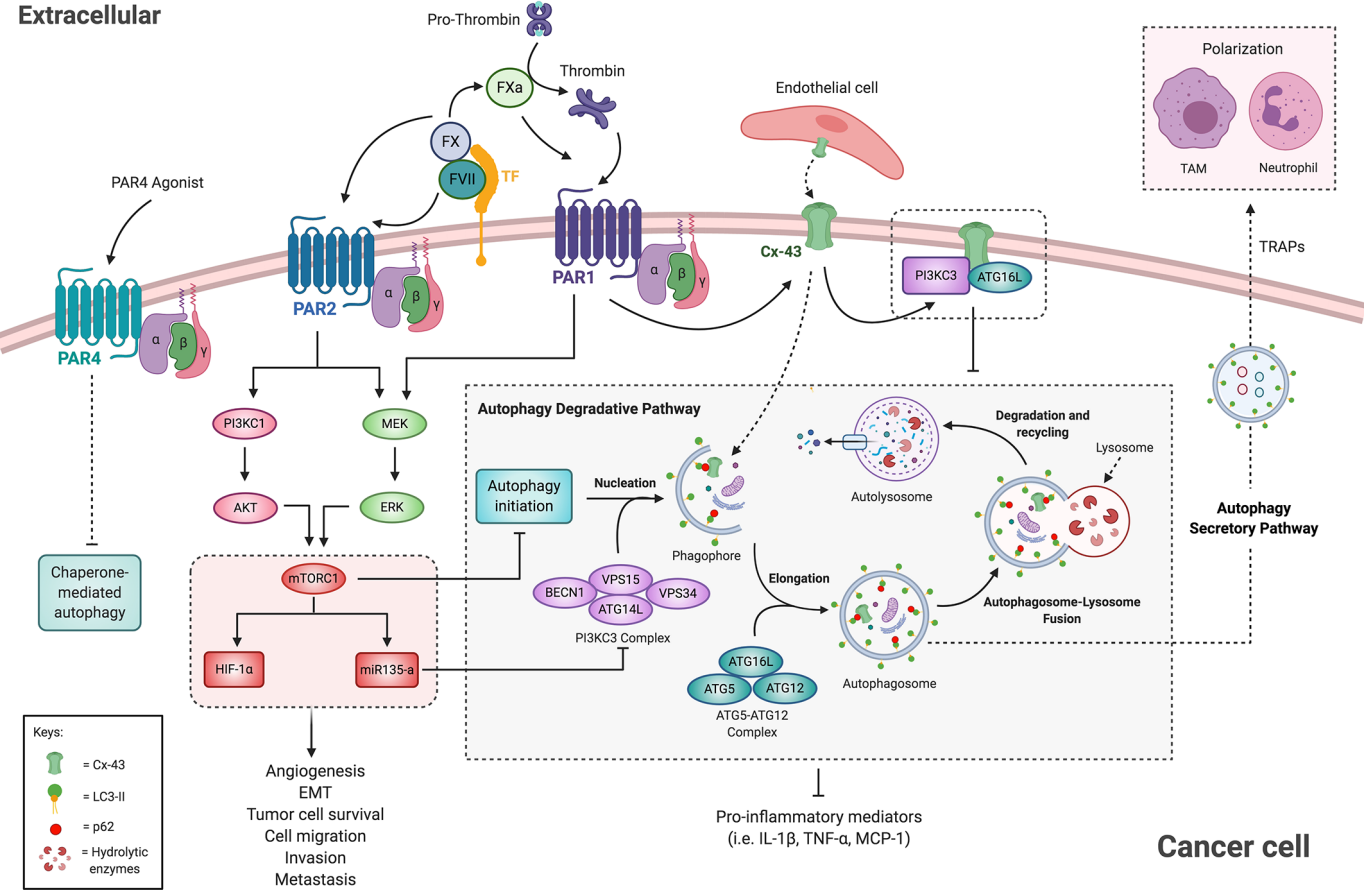
Crosstalk of the coagulation cascade and autophagy pathway in cancer cells. The activation of PAR1 and PAR2 by coagulation factors suppresses the autophagic pathway in an mTORC1-dependent manner. mTORC1 upregulates HIF1-α and miR135, both implicated in cancer and in autophagy regulation. PAR1 activation leads to Cx-43 up-regulation, which may impair autophagy by sequestering ATG16L and the PI3KC3 complex to the membrane. In turn, the autophagic pathway mediates PAR1 degradation. Additionally, PAR4 selective stimulation downregulates proteins associated with chaperone-mediated autophagy. Therefore, autophagy suppression by PARs activation leads to increased pro-inflammatory microenvironment and enhanced cancer progression and metastasis. Moreover, the autophagy secretory pathway participates in TRAPs release, which suppress anti-tumor immune response and thereby facilitate tumor progression. See the text for additional information. Cx-43, Connexin 43; EMT, Epithelial to Mesenchymal Transition; ERK, Extracellular-signal Regulated Kinase; FVII, Factor VII; FX, Factor X; FXa, activated FX; HIF1-α, Hypoxia-Inducible Factor-1; LC3, microtubule-associated protein 1A/1B-light chain 3; MEK, mitogen-activated protein kinase; MCP1, monocyte chemoattractant protein 1; Monocyte mTORC1, mammalian target of rapamycin complex 1; PAR, protease-activated receptor; PI3K, phosphatidylinositol 3-kinase; TAM, tumor-associated macrophage; TF, tissue factor; TRAPs, tumor cell-released autophagosomes.

As shown in **Figure 5**, thrombin mediated-PAR1 activation signals through PI3K/AKT/mTORC1 and activates Hypoxia-Inducible Factor-1 alpha (HIF-1α) (193), a well-known mediator of tumor survival, EMT, angiogenesis, and metastasis (193–195). Activation of PAR2 also leads to the downstream activation of the PI3K/AKT signaling pathway, promoting cell migration and invasion in both oral squamous and renal cell carcinoma (196, 197). In accordance, activation of both PI3K-AKT and mTORC1 signaling pathway by TF-FVIIa-Xa complex mediated by PAR1 and PAR2 has been associated with enhanced cell migration in a human breast cancer cell line (198, 199). Furthermore, recombinant TF, recombinant FVII or a PAR2 agonist upregulated mTORC1 signaling pathway in a hepatocellular carcinoma cell line (23). Moreover, levels of the autophagic marker LC3-II and the coagulation proteins TF, FVII and PAR2 were inversely correlated in human hepatocellular carcinoma tissues (23), suggesting a possible role of the coagulation pathway on autophagy suppression in cancer. In further accordance with an interaction between PAR signaling and autophagy, it was reported in human kidney tubular epithelial cells that PAR2 acting through the PI3K/AKT/mTOR pathway suppressed the process of autophagy, by affecting ATG5 and ATG12, and thus decreasing autophagosome formation (24). Additionally, downregulation of autophagy is associated with enhanced secretion of inflammatory mediators, such as IL-1β, TNF-α, and Monocyte Chemoattractant Protein-1 (MCP-1) (24). The aforementioned data suggest that activation of PAR2 by TF-FVII-Xa suppresses the autophagic pathway in a PI3K/AKT/mTOR-dependent manner (184), contributing to the generation of an inflammatory microenvironment that may lead to increased cancer cell migration and invasiveness. In this way, PAR signaling could directly promote tumor growth.

Interestingly, allograft models indicate that PAR1 promotes tumor growth by mediating immune escape. Tumor depletion of TF or PAR1 in allograft studies showed that CD8^+^ T cells effectively eliminated *Par1* KO cancer cells in immune-competent mice (21). Similarly, in a separate study, cancer cell derived-extracellular vesicles and tumor-released autophagosomes (TRAPs) mediated immune escape by T cell suppression. In particular, these vesicles induced autophagy and activation and polarization of neutrophils and macrophages into an anti-inflammatory phenotype that promoted tumor growth and immune escape in a Programmed Death-ligand 1 (PDL-1) dependent fashion (200, 201). Furthermore, blockade of autophagy in tumor cells promoted the switch of macrophages into the anti-tumor M1-like phenotype and restored immune function of tumor-infiltrating lymphocytes (TILs) (201). A tempting hypothesis would be that PAR signaling mediates the secretion of TRAPs through autophagy regulation, thus promoting secretory autophagy while decreasing degradative autophagy, which may promote both cancer cell survival and immune escape.

While extracellular vesicles may allow intercellular communications throughout the body, direct communication between tumor cells and ECs mediated by connexins may also be critical to tumor cell extravasation at a potential metastatic site (202). Connexin 43 (Cx-43) has been reported as an obstacle and a promoter of cancer progression. As examples, Cx-43 may obtain tumor suppressor gene status as its loss contributes to metastasis (203–205). Conversely, expression of Cx-43 has also been shown to enhance tumor metastasis through increased attachment and communication with the vascular endothelium (**Figure 5**) (206–209). In metastatic melanoma cell lines, PAR1 silencing decreased Cx-43 expression as well as cancer cell attachment to ECs and extravasation, suggesting that PAR1 contributes to invasion and metastasis *via* regulation of Cx-43 (210). However, it should be noted that most connexins are characterized by a rapid turn-over mediated by different degradation pathways, including autophagy, where p62 served as a cargo-recognition factor, forming a bridge between ubiquitinated Cx-43 and LC3, thereby leading it’s to degradation (211–213). In turn, connexins might negatively regulate the autophagic process at initial stages (214). As represented schematically in **Figure 5**, Cx-43 may recruit ATG16 and the PI3K-complex to the plasma membrane, limiting their availability and capacity for regulating autophagy (212). Thus, Cx-43 up-regulation induced by PAR1 could impair autophagy leading to enhanced migration and invasion in cancer.

Furthermore, PAR4 is also associated with cancer development (215), and its activation in esophageal squamous cell carcinoma (ESCC) leads to the downregulation of proteins associated with chaperone-mediated autophagy, such as the Heat shock protein family A (Hsp70) (216). This evidence strongly suggests that PARs activation mediates cancer progression by regulating signaling pathways associated with the autophagy process (**Figure 5**).

MicroRNAs (miRNAs) are now widely reported to play critical roles in the modulation of autophagy in cancer cells and as potential markers for cancer detection (217, 218). It is thus unsurprising that an association exists between coagulation-mediated autophagic suppression and tumor malignancies involving miRNAs participation (25). Huang and collaborators observed in hepatocellular carcinoma tissues that elevated levels of both miR-135a and FVII were associated with tumor stage, recurrence, microvascular invasion, and decreased disease-free survival (**Figure 5**) (25). Moreover, in Hep3B cells treated with recombinant TF, FVIIa, or a PAR2 peptide agonist, the expression levels of miR135a were increased. They further demonstrated that expression of this miRNA was dependent on mTOR levels and that miR-135a acts as a downstream effector of PAR2 activation, abrogating the autophagic process in an mTOR dependent manner (25). In the same line, in breast cancer cells, miR-142-3p inhibits autophagy by targeting HMGB1 (219), which can be delivered by platelets and is associated to autophagy in other cell types (220). Platelet-derived exosomes contain miR-126 (221), which inhibition enhances autophagy as determined by LC3-II increased and p62 decreased protein levels in ESCC (222). These few examples demonstrate that miRNAs secreted by the coagulation through the release of platelet exosomes may impinge on autophagy-related pathways and mediate cancer cell progression. Thus, future investigation should seek to evaluate the interplay between coagulation, autophagy and microRNA in the same biological models.

## Coagulation Cascade and Autophagy in the Tumor Microenvironment

The tumor microenvironment (TME) plays a principal role during tumor growth and metastasis. Within the tumor, ECs, immune cells and stromal cells alter their phenotype into a tumor-promoting state through secretion of growth factors and cytokines that sustain cancer cell survival and proliferation, while dampening the immune response. Interestingly, all these cells are subjected to stressors generated by cancer cells, such as glucose deprivation, hypoxia and inflammatory signaling; thus, autophagy plays a central role in the regulation of the TME. The TME is also a source of pro-thrombotic proteins, such as TF and FX, and thus in the subsequent section we will discuss how the interplay between the coagulation and autophagy cascades could maintain and promote tumor growth.

### Coagulation Cascade and Autophagy in the Tumor Endothelium

**Figure 6:**
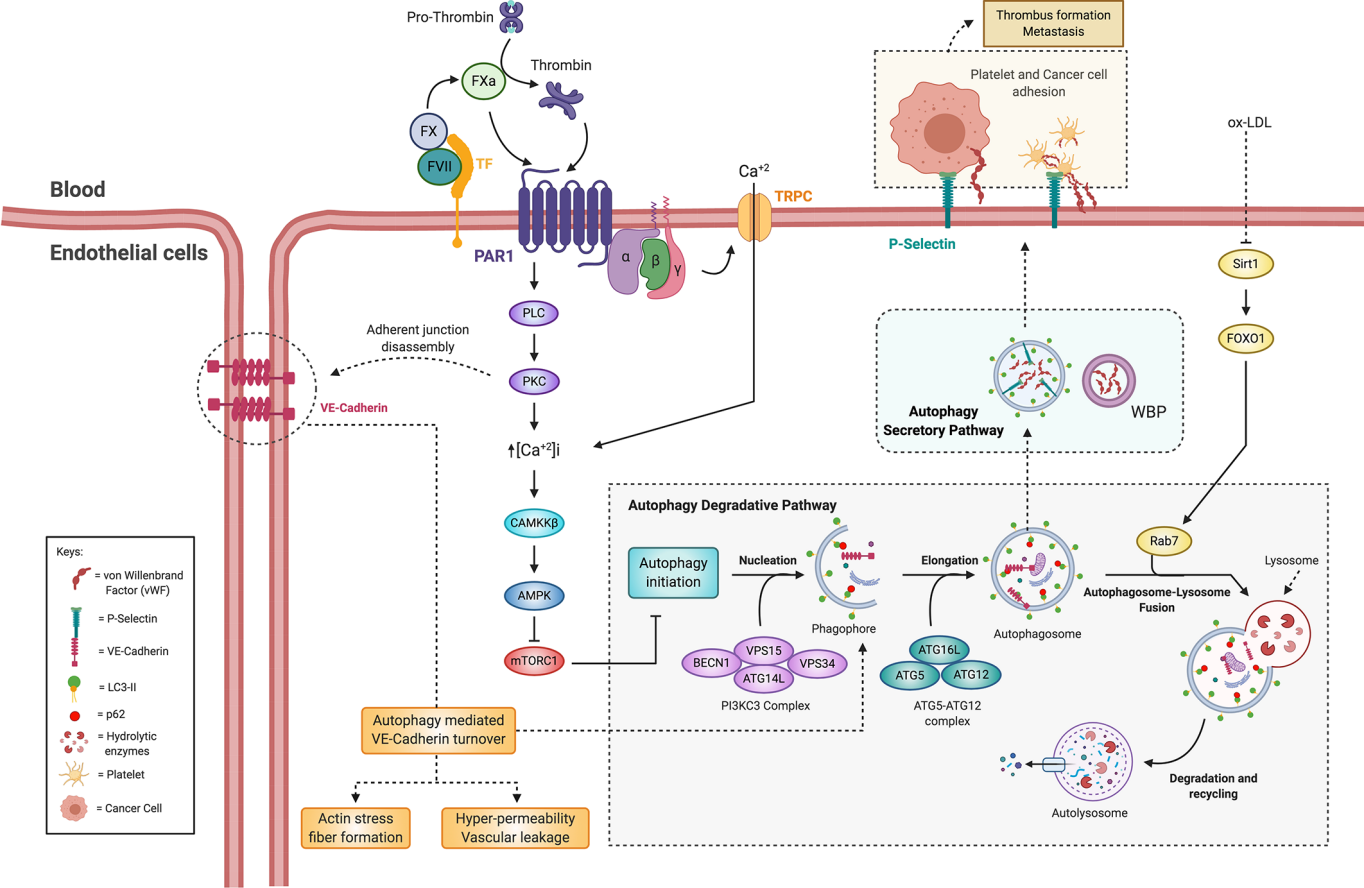
Crosstalk between the coagulation cascade and autophagy on tumor-associated endothelial cells. Thrombin-induced endothelial hyperpermeability and endothelial secretion of thrombosis-promoting factors are dependent on autophagy. Activation of the coagulation cascade and PAR1 induces an increase in intracellular Ca^+2^, which allows activation of AMPK by CaMKKB. Thus, the activation of AMPK induced by PARs could induce autophagy. Disassembly and degradation of VE-cadherin and the formation of actin stress fibers promoted by thrombin through autophagy could allow endothelial hyperpermeability. The secretion of vWF and P-selectin is mediated by the secretory pathway autophagy. Autophagosomes contain vWF and allow its release from endothelial cells. The secretion of vWF and P-selectin stimulated by ox-LDL is mediated by the inhibition of Sirt1/FoxO1 signaling by preventing fusion of autophagosome with lysosome. These factors allow adhesion of cancer cells and platelets to the endothelium, thus promoting cancer-related thrombosis and metastasis. FVII, factor VII; FX, factor X; FXa, factor X activated; TF, tissue factor; PAR1, protease-activated receptor 1; TRPC, transient receptor potential canonical; PLC, phospholipase C; PKC, protein kinase C; CaMKKB, calcium/calmodulin-dependent protein kinase kinase B; AMPK, AMP-activated protein kinase; mTORC1, mammalian target of rapamycin complex 1; VE-cadherin, vascular endothelial cadherin; vWF, von Willebrand factor; WBP, Weibel-Palade bodies; Sirt1, sirtuin 1; FoxO1, forkhead box protein O1; ox-LDL, oxidized low-density lipoprotein.

During metastasis, cancer cells acquire an invasive phenotype and detach from the primary tumor and enter the bloodstream in a process called intravasation. Subsequently, cancer cells migrate out of the bloodstream by the process of extravasation and establish metastatic foci at distant organs (223). Fundamental to both these processes is endothelial permeability, which is promoted by the coagulation factors thrombin and FXa (15, 224). Interestingly, recent studies have shown that autophagy is involved in thrombin-induced endothelial dysfunction. Thrombin promotes VE-cadherin disassembly and degradation, allowing endothelial hyper-permeability in through BECN1 (225). Likewise, *in vitro* knockdown of *Atg5* inhibited thrombin-induced actin stress fiber formation and VE-cadherin loss at the cell surface, thus preventing endothelial barrier dysfunction (226). Furthermore, pharmacological inhibition of autophagy with 3-MA, BafA1, or CQ can abrogate thrombin-induced hyperpermeability (227). Thus, in response to thrombin, VE-cadherin degradation through autophagy may lead to vessel hyperpermeability, as shown in **Figure 6**.

Poor vascular integrity contributes to the TME. Thrombin induces macrophage migration Inhibitory Factor (MIF) secretion from ECs, and the process of autophagy is involved in MIF-mediated endothelial hyperpermeability. Chao and colleagues showed that blocking autophagy attenuated thrombin-induced hyperpermeability in EC lines. Furthermore, blocking of autophagy or MIF effectively alleviated vascular leakage (227). These data suggest that endothelial permeability modulated by coagulation factors is dependent on autophagy (**Figure 6**), although further studies are required to evaluate these processes in the context of cancer.

How coagulation factor signaling pathways interact with autophagy is still an open question. However, upon ECs we know that thrombin binding to PAR1 initially induces Ca^+2^ mobilization through activation of the Gq/11-phospholipase C pathway and members of the transient receptor potential canonical (TRPC) family of channels (228, 229). This raise in [Ca^2+^]i activates CaMKKβ, which in turn, activates AMPK (228, 230, 231). Hence, it is tentative to suggest that activation of PARs by coagulation factors induce AMPK activation and thus initiate autophagy (**Figure 6**).

Within ECs, the autophagy machinery is associated with vWF and P-selectin secretion in autophagic vacuoles (106, 232, 232). The primary function of vWF is to create a cell-surface adhesion site for coagulation factor VIII or platelet adhesion (among other proteins) at the endothelium membrane. Upon vascular damage, secretory granules called Weibel-Palade Bodies (WPBs), which contain thrombosis promoting factors, are assembled into chains that bind to adjacent connective tissue and in turn trap circulating platelets (234). Notably, Torisu et al. showed that autophagosomes contain vWF and that pharmacological inhibition of the autophagy or knockdown of *Atg5* or *Atg7* inhibits vWF secretion (106). Accordingly, EC-specific KO of *Atg5* or *Atg7* increased mice bleeding time (106). However, the size and composition of thrombus did not vary in EC specific *Atg5*-KO mice (235). Moreover, the secretion of vWF and P-selectin in response to the ox-LDL pro-thrombotic stimulus is associated with a decrease in Sirt1/FoxO1 signaling, and therefore autophagic flux. Moreover, the increased release of vWF and P-selectin is mediated by the inhibition of the Sirt1/FoxO1 pathway that depresses the fusion of the autophagosome with the lysosome, thus favoring the secretion of these factors (**Figure 6**) (232).

Interestingly, it has been observed in cervical and breast cancer cell lines that decreased autophagosome-lysosome fusion is associated with increased release of extracellular nanovesicles, suggesting that extracellular secretion might provide a supplementary pathway to maintain cellular homeostasis when the autophagy degradative pathway is damaged (109). Moreover, extracellular vesicle secretion may contribute to tumor progression and metastasis (110, 111). Indeed, exosome secretion is shown to be essential for directional and efficient migration of HT1080 fibrosarcoma cells (236). Thus, these examples suggest a possible crosstalk between degradative and secretory autophagy pathways to maintain cellular homeostasis and tumor cell survival (99).

Within the TME, endothelial autophagy regulation of coagulative factors may contribute to cancer progression and cancer-related thrombosis. Cancer cells promote the secretion of WPBs and vWF from ECs (237, 238) and elevated plasmatic vWF correlate with tumor grade and metastasis (239). Moreover, vWF secretion has been reported to contribute to the process of EMT (240), the secretion of pro-inflammatory cytokines, and vascular permeability (234). Several studies show vWF-dependent cancer cell adhesion to the endothelium is mediated by integrin receptors and facilitates extravasation during metastasis (239, 241–243). It is tempting to speculate that autophagy plays a central role in mediating cancer-related thrombosis and metastasis by regulating vWF release from the activated endothelium.

Autophagy also possesses a role in the constitutive recycling of PAR1, a pivotal process to maintain the receptor pool and enable re-sensitization to its potential coagulation cascade agonists. Rab11A and Rab11B are involved in autophagosome formation by regulating membrane transport from recycling endosomes (52). Grimsey and collaborators found that under basal conditions PAR1 is constitutively internalized and recycled back to the cell surface by a Rab11B-dependent pathway, whereas Rab11A regulates PAR1 basal lysosomal degradation (244). Interestingly, when recycling is disrupted in Rab11B-deficient cells, PAR1 is sorted from endosomes to autophagosomes and subsequently degraded in autolysosomes, in a Rab11A and ATG5 dependent manner (244). These results support a role for Rab11A in PAR1 basal autophagosomal-lysosomal sorting. Consistently, in EC cell lines, Rab11-B depleted cells showed decreased expression of PAR1, as a sign of its increased degradation. Conversely, in Rab11A deficient ECs, PAR1 protein expression was elevated. Thus, Rab11B and Rab11A serve distinct functions and regulate PAR1 recycling or basal autophagic/lysosomal degradation, respectively (244).

Autophagy may regulate the signaling pathways of different coagulation factors and their appropriate cellular responses in ECs by altering the recycling and endosomal sorting of PAR1 as well as in pro-thrombotic factors release. Furthermore, the pro-inflammatory, hypoxic and nutrient-starved state of the TME may deregulate EC autophagy, promoting coagulation and in turn paving the way for metastasis. Further experimental approaches should be applied to confirm this hypothesis.

### Coagulation Cascade and Autophagy Regulate Myeloid Cell Polarization Within the Tumor Microenvironment

Autophagy and inflammation work synergistically in the TME to facilitate tumor growth and metastasis (245). Within the TME, monocytes and macrophages are essential sources of extravascular FX and TF. Notably, the synthesis of FX myeloid cells determines the Tumor-Associated Macrophage (TAM) phenotype (20). Inhibition of FXa-PAR2 signaling causes reprogramming of TAMs and attenuates the recruitment of immunosuppressive neutrophils and regulatory T cells promoting anti-tumor immunity (20). Coagulation factors could facilitate invasion and metastasis by transforming monocytes and macrophages into TAM-like cells. In the same line, monocytes and macrophages treated with FXII exhibited polarized M2 phenotypes with up-regulation of CD163, IL-10, IL-8, CCL18, CCR2, and CXCR2 (246). It has been reported that FXII and FXIIa upregulate neutrophil functions, contributing to macrophage polarization and T-cell differentiation that may contribute to cancer progression [Reviewed in (247)]. Furthermore, epithelial ovarian cancer cells exposed to conditioned medium from FXII-stimulated monocytes/macrophages showed increased invasive potential (246). In the same way, TF-FVIIa complex produced CD14 and CD163 up-regulation in monocytes in addition to an increase in the expression of IL-10, IL-8, TNF-α, CXCR2, and CCR2 (248). Moreover, co-cultures of epithelial ovarian cancer cells with TF-FVIIa stimulated monocytes increased the invasive potential (248). Additionally, THP-1 human monocytic cell line stimulated with TF or FVIIa displayed an M2-like phenotype with high levels of IL-4, IL-10, TGF-β, and TNF-α. Gastric cancer cells co-cultured with TF-stimulated TAMs also showed increased migration and invasion (249). These TF-stimulated TAMs induced VEGF and MMP-9 expression, which could promote the invasive potential and angiogenesis (249). Furthermore, co-cultivation of TF-expressing cancer cell lines with human monocytes stimulated invasive capacity, an effect inhibited by a TF neutralizing antibody (250). In non-small cell lung cancer patients with lymph node metastasis, there are reported higher levels of monocyte TF mRNA, which correlate with overall survival (251).

Interestingly, Graf et al. showed that PAR2 signaling directly regulates TAM mediated immune-evasion (19). Given that macrophage-specific deletion of FX prevented *in vitro* macrophage polarization, these observations suggest that coagulation factors contribute to cancer progression by promoting the formation of TAM-like cells. Furthermore, TAM phenotypic changes were similar in both macrophage FX-deficient mice and PAR2 mutant mice and accompanied by increased T cell infiltration, suggesting that PAR2 activation by FXa impairs anti-tumor immunity (19, 20). FXa production through TF-FVIIa leading to PAR2 activation and the formation of the TF-FVIIa-FXa-Endothelial protein C receptor (EPCR) complex is essential in the TLR4-mediated innate immune responses [reviewed in (9)]. Moreover, the TF-FVIIa-FXa-EPCR complex selectively induces expression of the TLR3/4 signaling adaptor protein Pellino-1, the transcription factor interferon regulatory factor 8 (IRF8) and a set of interferon (IFN)-regulated genes (252).

Autophagy is also associated with macrophage phenotype and activation as the AKT pathway converges both inflammatory and metabolic signals (253). The activation of mTORC1 promotes M1 polarization (254) and in accordance autophagy modulates the activity of macrophages and their responses (255–257). In general, the induction of autophagy downstream of TLR/NF-κB promotes monocyte differentiation and M2 polarization [reviewed in (258)]. Since the coagulation cascade also interacts with TLR-NF-κB signaling promoting polarization toward an M2 phenotype, it would be interesting to evaluate if there is an interaction between autophagy and the coagulation cascade in terms of innate immune signaling within the TME.

### Coagulation Cascade, Autophagy, and Neutrophil Extracellular Traps in the Tumor Microenvironment and Cancer-Associated Thrombosis

**Figure 7:**
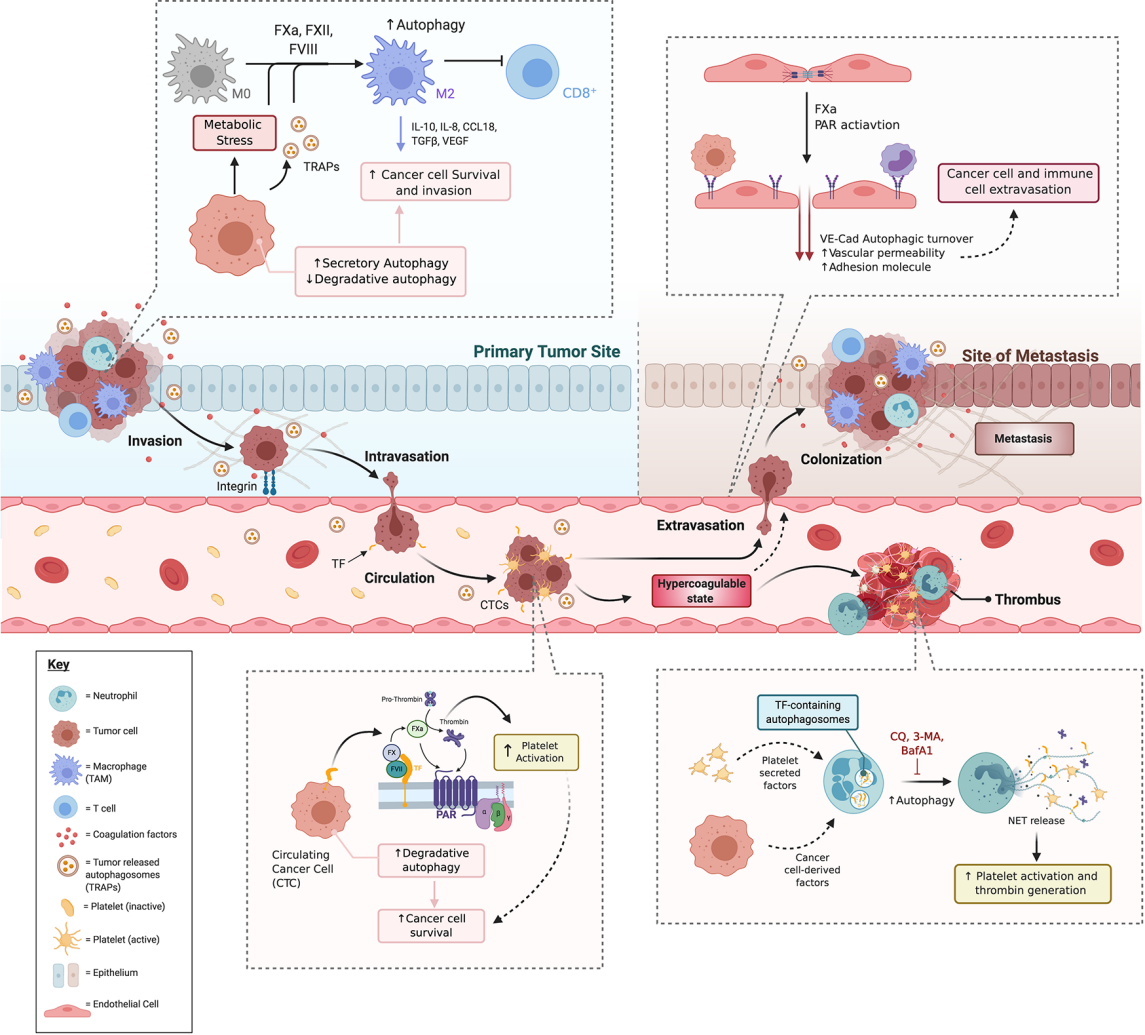
The crosstalk between coagulation and autophagy promotes cancer-associated thrombosis and metastasis. The coagulation system is an integral part of the multifaceted approach taken by the cancer cell to survive, propagate and ultimately exhaust the body’s capacity to function. We speculate that cancer cells may use common mechanisms to hijack immune cells, ECs, and platelets to integrate with the coagulation system for its own end. A common mechanism present in each of these components may be the hijacking of the autophagy pathway. At early stages, cancer cells downregulate degradative autophagy while they increase the secretion of TRAPs, indicating a disruption in the balance of degradative and secretory autophagy. This balance is influenced by the coagulation cascade, particularly, PAR activation. In turn, both TRAPs and PAR activation by coagulation factors FVII and FX promote macrophage polarization into a M2 phenotype (TAMs) with enhanced suppressive capacities, reducing cytotoxic T cell responses favoring tumor growth. PARs also promote EMT, thus enabling cancer cells to invade and intravasate. In circulation, CTCs imcrease degradative autophagy as a survival mechanism and can promote thrombogenesis through activation of the coagulation cascade on platelets, neutrophils and ECs leading to an hypercoagulable state, that also promotes metastasis through endothelial and neutrophil activation. BafA1, bafilomycin A1; CCL18, Chemokine (C-C motif) ligand 18; CQ, chloroquine; EMT, Epithelial to Mesenchymal Transition; FVII, Factor VII; FX, Factor X; FXa, activated FX; IL-8, Interleukin 8; IL-10, Interleukin 10; PAR, protease-activated receptor; TAM, tumor-associated macrophage; TF, tissue factor; TGFβ, Tumor Growth Factor beta; TRAPs, tumor cell-released autophagosomes; VE-Cad, Vascular Endothelial Cadherin; NET, Neutrophil Extracellular Trap; 3-MA, 3-methyladenine.

Neutrophils and platelets cooperate to enhance coagulation (259). The release of intracellular components by neutrophils promotes coagulation and this has been associated with hypercoagulability and cancer-related thrombosis (260). As clinically observed in patients with sepsis and deep vein thrombosis, the activation of platelets can induce neutrophil extracellular traps (NETs), which in turn potentiate platelet aggregation (261–263). Once again demonstrating a connection to thrombosis and cancer, NETs can also formed under conditions of inflammation (264–267). As schematized in **Figure 7**, neutrophils can translocate TF to the generated NETs, where neutrophil elastase enhances TF pro-thrombotic activity through the degradation of TFPI (9). Elastase is capable of activating PAR1 and PAR2 receptors (268, 269), which may contribute to thrombus formation by promoting platelet and EC adhesion and activation.

Interestingly, the autophagy pathway is involved in mediating the delivery of thrombogenic TF to NETs, thus promoting thrombin generation and subsequent PAR-1 signaling (270). Moreover, in neutrophils, the inclusion of TF in autophagosomes is associated with its extracellular delivery. Disruption of autophagy by addition of 3-MA or BafA1 can abrogate NET release and TF trafficking, respectively (270). Notably, autophagy-mediated NET formation has been associated with CAT (271, 272), suggesting a potential interaction between autophagy, neutrophils and platelets within TME.

In line with this hypothesis, tumor-bearing mice incapable of forming NETs display decreased platelet aggregation and decreased circulating TF (271). Moreover, under in both *in vitro* and *ex vivo* experimental conditions, the pre-treatment of neutrophils with CQ inhibited NET formation (273, 274). TF, located in acidified autophagosomes, is released into NETs upon neutrophils exposure to inflammatory stimuli. Furthermore, TF from NETs can induce both thrombin generation and platelet activation mediated by PAR1 signaling (270). These data suggest that the release of TF through NETs could cause localized activation of the coagulation cascade and subsequent PAR dependent activation of platelets and ECs.

Interestingly, neutrophils from tumor-bearing orthotropic mice have increased LC3-II expression (274). Inhibition of autophagy with CQ or genetic ablation of the Receptor for Advanced Glycation End-products (RAGE), a Class III MHC protein receptor that mediates autophagy, resulted in reduced NET formation frequency within the TME (274). Based on these findings, Boone et al., proposed that NETs are upregulated in pancreatic cancer through RAGE (274). In the same line of thinking, a separate study found that, in response to platelet-derived microparticles, neutrophils can increase autophagy, mobilization of the granule content, enhanced proteolytic activity, prolonged survival, and generation of NETs (275). Neutrophil autophagy and the generation of NETs were also blunted in the presence of a competitive inhibitor of the cytokine mediator of inflammation HMGB1 (275). These findings are in line with other studies showing that autophagy in the neutrophil is essential for the NET formation, and this process is deregulated in cancer.

## Targeting the Coagulation Cascade and Autophagy for Cancer Treatment

How can the knowledge of the interplay between coagulation and autophagy be put in practice in the oncology clinic? In truth, the axis of TF-FXa/thrombin-PAR-AKT- mTOR is already exploited in numerous treatments such as the rapamycin family of drugs, anticoagulants, metformin and certain targeted therapies. VTE is a significant cause of cancer-related death (276) and its prophylactic treatment reduces mortality, with low molecular weight heparin (an anticoagulant) often employed as the first-line treatment (7). As discussed previously, anticoagulants such as low molecular weight heparin, Rivaroxaban and Dalteparin have also demonstrated direct effects on tumor growth, immune-evasion and metastasis. It has already been speculated that the specific targeting of FXa could reduce metastasis and promote anti-tumor immunity (15, 20). It was previously reported that exogenous FXa increases melanoma metastasis to the lung, spleen and lymph nodes, together with an accumulation of intra-peritoneal fluid in a syngeneic mice model (15). Furthermore, the co-administration of the anticoagulant Dalteparin reduced the FXa- increased lung metastasis, while no metastasis was observed in other organs (15). A separate study demonstrated that the targeted deletion of this coagulation factor in myeloid cells reduced tumor progression in animal models, adding justification for pharmaceutical intervention in this pathway.

Furthermore, the anticoagulant Rivaroxaban has been demonstrated to give similar results to that of anti–PD-L1 therapy, and these two treatments were shown to synergize to improve anti-cancer immunity. As a mechanism, the authors propose that FXa signals through PAR2 to promote immune evasion, an effect attenuated by Rivaroxaban through the reprogramming of TAMs (20). Furthermore, co-treatment with a thrombin inhibitor, Dabigatran, and cisplatin in a model of ovarian cancer reduced tumor growth and levels of circulating activated platelets compared to Dabigatran or cisplatin alone. Interestingly, these authors demonstrated that this co-treatment with Dabigatran promoted anti-tumor activity of cisplatin by alleviating the immunosuppressive microenvironment. Co-treatment significantly decreased the number of Myeloid-Derived Suppressor Cells (MDSCs) and dendritic cells while increasing IFN-γ production by CD8^+^ effector T cells in ascites (277).

Similarly, in a melanoma metastasis model FXa increases endothelial permeability and promotes immune infiltration into mouse lungs, and this accumulation was reduced by the presence of the anticoagulant Daltepatin (15). These results further support the use of direct oral anticoagulants to reduce metastasis and favor an immune response against the tumor. Of note is that the inhibition of FXa on metastasis may be distinct to that of thrombin, shedding further light on the non-coagulation related roles of these coagulation factors. These observations also highlight the possibility that compounds may be developed that differentially inhibit either the coagulation or the non-coagulation actions of coagulation factors.

PARs, central proteins in the pathway connecting coagulation and in malignancy, are already a recognized cancer target (278). TF-mediated signaling *via* PAR2 has been associated with proliferation, migration and invasion of the cancer cell, and accordingly, the use of an anti-TF antibody has been shown to block PAR2 activation and suppresses tumor growth while demonstrating minimal effects on the coagulation process (279, 280). PAR inhibitors like vorapaxar, atopaxar, and PZ-128 have undergone clinical evaluation in the oncology setting. Evidence from metastatic breast cancer suggests that PAR1 blockade with PZ-128 in combination with Taxotere could be beneficial. Furthermore, a benefit may be present in dual-standard chemotherapy regimens and PZ-128 treatment in breast and ovarian cancers (278).

A further potential therapeutic target is oxidoreductase-protein disulfide isomerase (PDI) that catalyzes a thiol-disulfide exchange. In monocytes and macrophages, the activation of the TF cascade is reported to require a thiol-disulfide exchange and PDI (9). Liberated from both the EC and platelets upon agonist vascular injury, the subsequent use of PDI inhibitors could potentially attenuate platelet thrombus formation and fibrin deposition (9). Inhibition of PDI, by either antibodies or a non-specific thiol inhibitor decrease thrombus formation and fibrin generation in a mouse model of thrombosis and thus it will be interesting to examine if this could give potential benefit in the cancer setting (281).

As a central pathway for cellular function, the autophagy pathway is also a potential target for therapeutic intervention in cancer. To this end, the understanding of the action of rapamycin, discovered from a plant on Easter Island, has led to a series of “rapalogs” entering clinical oncology practice. Rapamycin, a specific mTOR inhibitor, can decrease cell migration promoted by the formation of TF-FVIIa-FXa complex in breast cancer cell model (199). Rapamycin, in combination with doxorubicin, can bring about remission in an AKT-positive lymphoma mouse model by blocking AKT signaling and overcoming chemotherapy resistance (282). Furthermore, in a vascular malformational model, the levels of D-dimer, a direct indicator of coagulation, were significantly decreased following treatment with an mTOR inhibitor (283).

Interestingly, long non-coding RNAs have been suggested to be a mechanistic target of mTOR signaling, ubiquitin-mediated proteolysis, and the coagulation cascade, opening the door to future intervention with RNA targeted therapy in cancer (284). However, as with any distribution of a ubiquitous process in cellular machinery, the use of mTOR inhibition may bring caveats. In an EC model, rapamycin strongly enhances the VEGF-induced expression of TF, possibly due to the interference in the negative feedback mechanism controlling this cycle. As VEGF is upregulated in tumors, this may explain the tumor vessel thrombosis observed in patients undergoing rapamycin therapy (285). Understanding the signaling of the coagulation system and its interaction upon cancer cell progression will be necessary for the targeted selection by the plethora of anticoagulants currently on the market to reduce tumor burden without perturbing essential hemostatic signaling.

Precision medicine based on Tyrosine Kinase Inhibitors (TKIs) has been reported to induce autophagy in many types of cancer cells (286). Noteworthy, the combination of certain TKIs with azithromycin (which dampens autophagy), enhanced cytotoxicity (286, 287). In the same line, an antibody-modified nanoparticle containing a combination of gefitinib and CQ was shown to have potential benefit in overcoming acquired EGFR-TKI resistance (288). Similarly, a combination of BafA1 with Gefitinib improved anti-tumor activity in a mouse model of triple-negative breast cancer (289). This burgeoning field may show the potential of multi-targeting to achieve both tumor-targeting selectivity and autophagy inhibition.

As previously discussed, recent studies have brought to light the immune regulatory role of coagulation components in the TME. FXa promotes immune evasion by signaling through PAR2, and the consequent addition of Rivaroxaban reprograms TAMs, supporting the translational potential of direct oral anticoagulants to overcome resistance to immunotherapy. A recent publication reported a before unidentified role of the immune checkpoint Programmed cell death protein 1 (PD-1) in regulating both lineage commitment and cell metabolism in cancer-associated myelopoiesis (290). These authors demonstrated that myeloid progenitors deficient for PD-1 manifested enhanced activation of mTORC1 in response to Granulocyte-Colony Stimulating Factor (G-CSF). This myeloid cell-specific ablation of PD-1 increased T memory cell function and anti-tumor activity. This evidence opens the door to the potential manipulation of the autophagy pathway during cancer immunotherapy.

### Conclusion

The coagulation cascade and specifically its primary initiator TF possess effects that extend well beyond hemostasis and into the poorly characterized cauldron of cancer progression. Furthermore, the PARs, once believed to be exclusive mediators of the thrombin activation of platelets, have now manifested their versatility in regulating the intracellular pathways of almost every cell type examined. At the center of every cell lies the machinery for the process of autophagy, another black hole of information, yet this cascade is known to impinge on every biological process imaginable to the minds of cell biologists and physiologists. A NCBI Pubmed search in late 2020 with the keywords coagulation, PAR, autophagy and cancer will bring back only a handful of publications. However, each word alone will offer up more references than the most diligent biomedical investigator could read in their lifetime. Therefore, it is evident that we are extensively researching each integral pathway that classically appears in our physiology textbooks. However, there is little integration between the disciplines. In this review, we made a first attempt to examine coagulation and cancer signaling pathways to try and find common ground between each cascade and in particular, identify if autophagy is at the center of the intersections. Herein, we illustrated that each pathway has components in common.

Although the functional relevance of autophagy in cancer requires further study, it is proposed that autophagy acts as a protective mechanism during cancer initiation, yet promotes later stage tumor growth and metastasis. Evidence that autophagy-associated cell death acts as an initial tumor suppressor comes from the observation that many tumors present deletions in autophagy-related genes; moreover, loss of autophagy can induce genomic instability. Conversely, autophagy could promote cancer cell survival under metabolic stress, thereby facilitating metastasis by promoting cancer cell survival. The later hypoxic, nutrient-starved and pro-inflammatory TME may deregulate local and distant autophagic pathways in ECs, platelets, immune cells, and HSCs promoting coagulation and paving the way for metastasis and thrombosis. Moreover, the knowledge that components of the autophagy machinery are required for non-conventional protein secretion of pro-inflammatory and thrombotic mediators into the TME suggests that not only conventional autophagy but also secretory pathway may have a role in CAT and metastasis. Hopefully, this first in-depth analysis of the crossovers in these differing pathways will serve to bring to light possible new areas of investigation and elucidate strategies for future therapeutic intervention.

## Author Contributions

All authors contributed to the article and approved the submitted version.

## Funding

This study was funded by the grants issued by the Government of Chile: CONICYT FONDAP-15130011 (GO), Millennium Institute on Immunology & Immunotherapy IMII P09/016-F (GO), FONDECYT 1180241 (GO). The first author (CH) is a doctoral scholarship recipient of the Vice-rectory of Investigation of the Pontifical Universidad Católica de Chile. The authors (MH-C) and (CA) are recipients of CONICYT PhD fellowships (N°21151609 and 21201596, respectively). All figures were created with BioRender.com.

## Conflict of Interest

The authors declare that the research was conducted in the absence of any commercial or financial relationships that could be construed as a potential conflict of interest.
